# Supplementary material for: Chimpanzee culture beyond the conspicuous: Evidence for broad-scale observational social learning in wild individuals
Source: iScience. 2026 Apr 28;29(6):115922. doi: 10.1016/j.isci.2026.115922 (PMC13214372; doi:10.1016/j.isci.2026.115922)
Supplement: Document S1. Figures S1–S9, Tables S1–S8, and supplemental references [file mmc1.pdf]

iScience, Volume *29*

Supplemental information

Chimpanzee culture beyond the conspicuous: Evidence for broad-scale observational social learning in wild individuals

Nora E. Slania, Mariana Gómez-Muñoz, Ayrin-Sophie Piephoh, Geresomu Muhumuza, Richard Young, T. Revathe, Catherine Hobaiter, Klaus Zuberbühler, and Caroline Schuppli

Model A: Effects of focal age, food item frequency, and food item complexity on peering frequencies

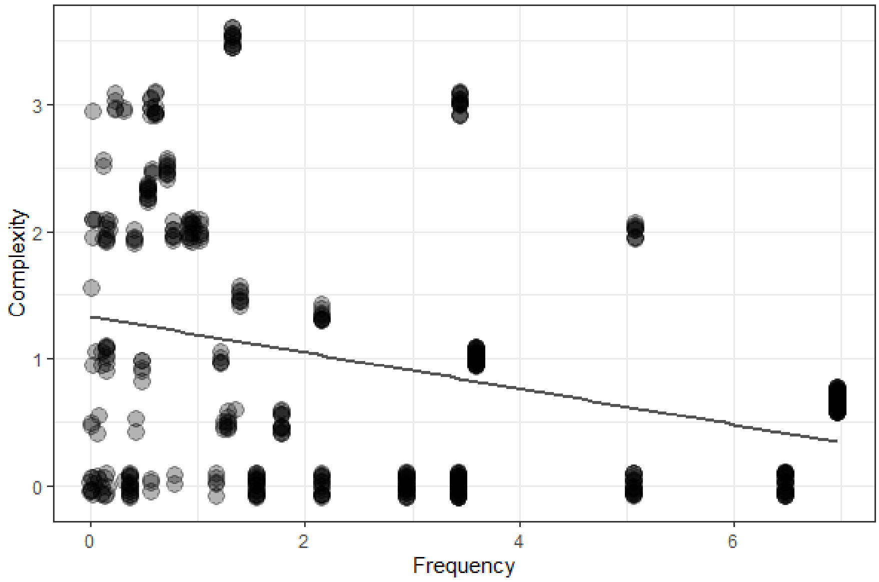

Figure S1: Distribution of food items by frequency and complexity. Related to STAR Methods Model A.

Table S1: Model A output, related to Figure 2. Model estimates and respective standard error, credibility intervals (Q2.5-Q97.5), Rhats, Bulk ESS and Tail ESS. All predictors ("complexity", "frequency", "age", "age^2") were z-transformed. Credibility intervals not comprising zero are marked in bold.

	Estimate	Est.Error	Q2.5	Q97.5	Rhat	Bulk_ESS	Tail_ESS
Intercept	-1.438	0.458	**-2.394**	**-0.597**	1	7276.509	8661.897
comp_z	0.642	0.209	**0.249**	**1.075**	1	5512.26	7039.836
freq_z	-0.696	0.299	**-1.269**	**-0.087**	1	6062.381	7679.016
age_z	-3.252	0.975	**-5.374**	**-1.606**	1	6066.579	6153.285
age_z2	-3.165	1.094	**-5.469**	**-1.194**	1	5421.948	6007.998

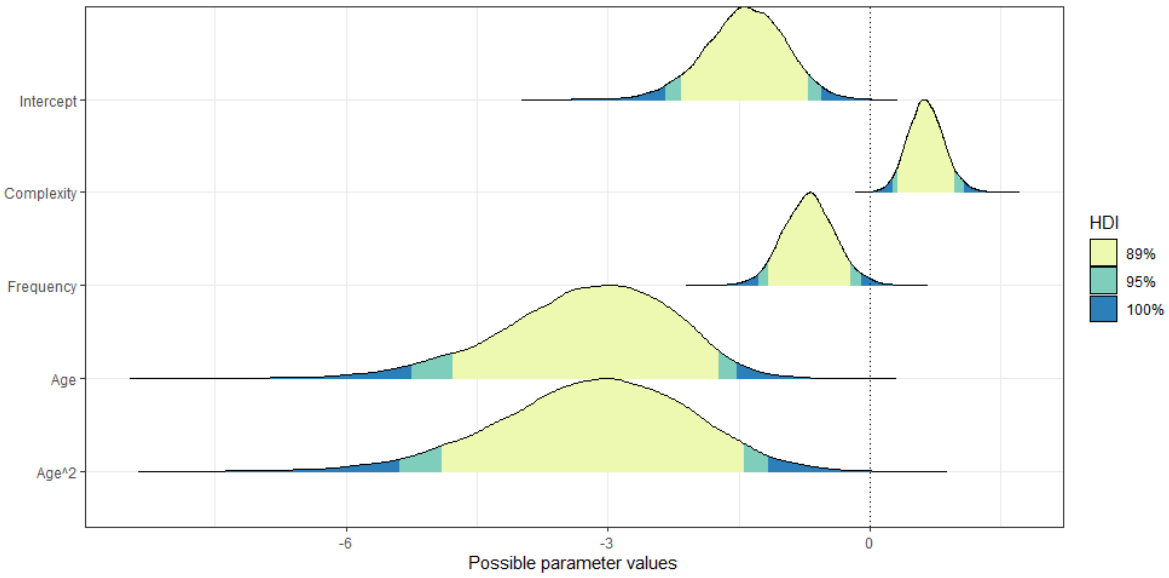

Figure S2: HDI of Model A, related to Figure 2. HDI are shown for each model estimate with credibility intervals of 89% marked in yellow, of 95% marked in green, and of 100% marked in blue. All model predictors were z-transformed.

Table S2: Supplementary Model A.1 output, related to STAR Methods Model A. Model estimates and respective standard error, credibility intervals (Q2.5-Q97.5), Rhats, Bulk ESS and Tail ESS. All predictors ("complexity", "frequency", "age", "age^2") were z-transformed. Credibility intervals not comprising zero are marked in bold.

	Estimate	Est.Error	Q2.5	Q97.5	Rhat	Bulk_ESS	Tail_ESS
Intercept	-1.448	0.466	**-2.426**	**-0.599**	1.001	10093.531	9659.126
comp_z	0.737	0.33	**0.113**	**1.415**	1	8415.279	8225.436
freq_z	-0.685	0.31	**-1.291**	**-0.057**	1	9775.662	8926.204
age_z	-3.289	1.004	**-5.481**	**-1.598**	1	9490.216	7153.911
age_z2	-3.213	1.13	**-5.644**	**-1.208**	1	9020.363	6985.53
comp_z: freq_z	0.121	0.344	-0.549	0.821	1	8971.779	9114.571

Table S3: Supplementary Model A.2 output, related to STAR Methods Model A. Model estimates and respective standard error, credibility intervals (Q2.5-Q97.5), Rhats, Bulk ESS and Tail ESS. All predictors ("complexity", "frequency", "age", "age^2") were z-transformed. Credibility intervals not comprising zero are marked in bold.

	Estimate	Est.Error	Q2.5	Q97.5	Rhat	Bulk_ESS	Tail_ESS
Intercept	-1.717	0.506	**-2.775**	**-0.804**	1	6444.141	8128.623
comp_z	0.828	0.339	**0.18**	**1.51**	1	4813.061	7484.446
age_z	-3.95	1.186	**-6.697**	**-2.038**	1.001	4030.568	4661.856
age_z2	-3.563	1.203	**-6.245**	**-1.543**	1.001	4022.643	5005.549
freq_z	-0.818	0.444	-1.725	0.028	1	5217.621	6586.852
comp_z: age_z	0.065	0.927	-1.717	1.939	1.001	4142.497	4919.346
comp_z:	-0.429	0.937	-2.242	1.435	1.001	4510.801	5338.179

age_z2							
age_z: freq_z	0.085	1.027	-2.149	1.963	1	4102.082	4153.779
age_z2: freq_z	0.419	1.015	-1.718	2.316	1	4208.113	4682.544

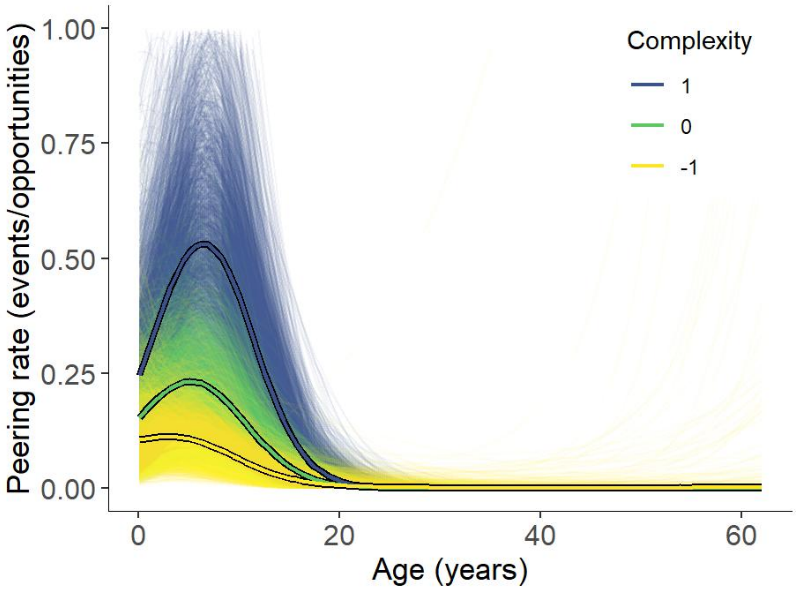

Figure S3: Peering frequencies at foot item complexity, related to Figure 2. Relative peering frequencies at different food item complexities (z-transformed mean complexity +/- SD) over age, using model estimates from Supplementary Model A.2.

Model B: Effect of target age on peering frequencies across development

Table S4: Model B output, related to Figure 3. Model estimates and respective standard error, credibility intervals (Q2.5-Q97.5), Rhats, Bulk ESS and Tail ESS; continuous predictors (age, age^2) were z-transformed; categorical predictor age relation was dummy coded (reference category age mate). Credibility intervals not comprising zero are marked in bold.

	Estimate	Est.Error	Q2.5	Q97.5	Rhat	Bulk_ESS	Tail_ESS
Intercept	-0.748	0.255	**-1.245**	**-0.248**	1	11720.383	10286.339
age_z	-1.792	0.528	**-2.944**	**-0.879**	1	6343.841	5923.332
age_z2	-1.554	0.535	**-2.695**	**-0.575**	1.001	5442.449	5068.717
AgeRelation Older	-12.065	8.947	**-35.422**	**-1.601**	1.001	2839.917	1251.669
AgeRelation Younger	-2.759	0.485	**-3.748**	**-1.85**	1	12536.334	8871.882
age_z: AgeRelation Older	-45.758	36.979	**-144.207**	**-4.069**	1.002	2721.098	1205.081
age_z: AgeRelation Younger	0.992	0.82	-0.582	2.637	1	8818.4	8384.312
age_z2: AgeRelation Older	-45.959	37.26	**-146.188**	**-6.399**	1.001	2629.275	1255.782
age_z2: AgeRelation Younger	1.644	0.613	**0.502**	**2.92**	1.001	6003.043	4452.946

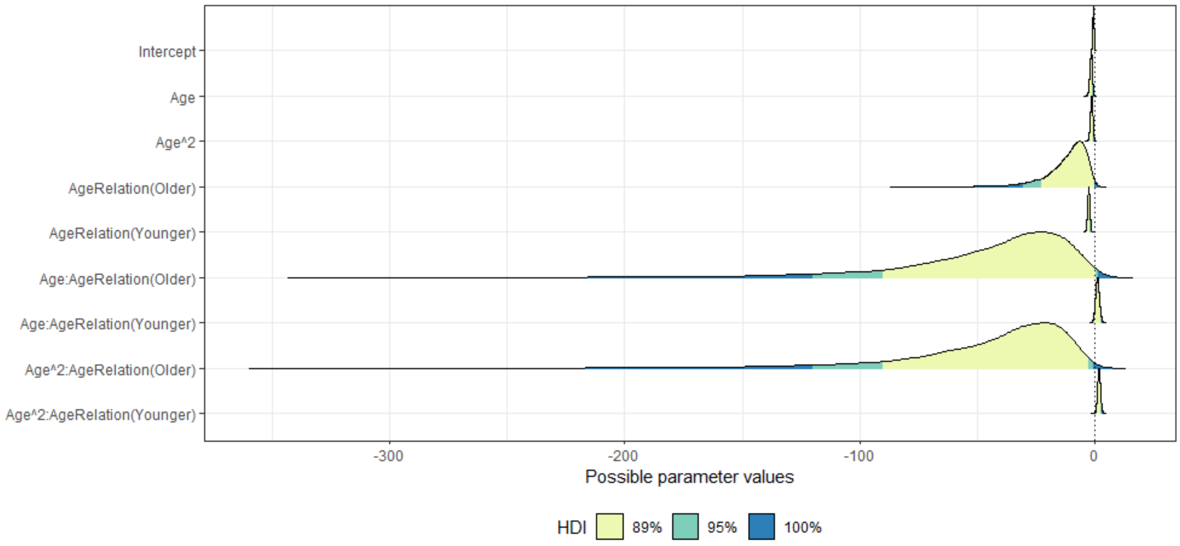

Figure S4: HDI Model B, related to Figure 3. HDI are shown for each model estimate with credibility intervals of 89% marked in yellow, of 95% marked in green, and of 100% marked in blue; continuous predictors (age, age^2) were z-transformed; categorical predictor age relation was dummy coded (reference category age mate).

Model C1: Effect of kin relation on overall peering frequencies

Table S5: Model C1 output, related to Figure 4. Model estimates and respective standard error, credibility intervals (Q2.5-Q97.5), Rhats, Bulk ESS and Tail ESS; continuous predictors (age, age^2) were z-transformed; categorical predictor kin relation was dummy coded (reference category unrelated). Credibility intervals not comprising zero are marked in bold.

	Estimate	Est.Error	Q2.5	Q97.5	Rhat	Bulk_ESS	Tail_ESS
Intercept	-1.695	0.336	**-2.393**	**-1.062**	1.001	5025.157	6785.462
age_z	-0.038	0.4	-0.873	0.721	1.001	6487.885	6729.156
age_z2	-0.376	0.243	-0.858	0.109	1	7338.591	7270.745
target Mother	-2.793	0.684	**-4.283**	**-1.575**	1	6297.892	6323.216
target Kin	-1.19	0.388	**-1.97**	**-0.443**	1.001	9634.322	9811.204
age_z: target Mother	-5.983	2.769	**-12.196**	**-1.63**	1	4553.29	4596.03
age_z: target Kin	-3.445	0.983	**-5.718**	**-1.914**	1.001	6703.59	5400.005
age_z2: target Mother	-4.783	2.798	**-10.939**	**-0.196**	1	4888.903	5092.619
age_z2: target Kin	-0.448	0.997	-2.786	1.025	1	7301.568	6047.182

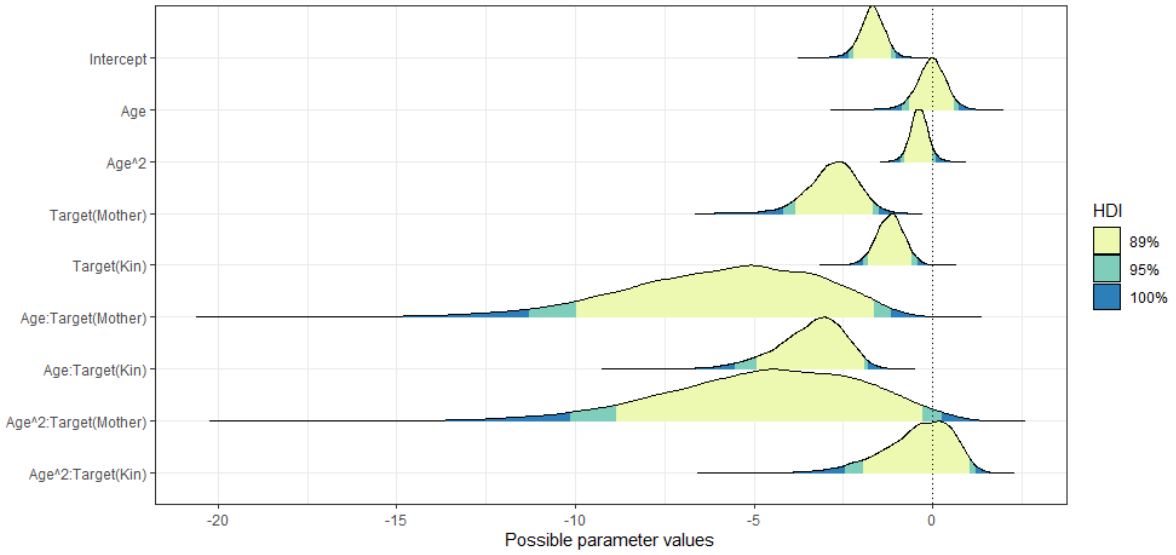

Figure S5: HDI of Model C1, related to Figure 4. HDI are shown for each model estimate with credibility intervals of 89% marked in yellow, of 95% marked in green, and of 100% marked in blue; continuous predictors (age, age^2) were z-transformed; categorical predictor kin relation was dummy coded (reference category unrelated).

Model C2: Effect of kin relation on peering opportunity use

Table S6: Model C2 output, related to Figure 4. Model estimates and respective standard error, credibility intervals (Q2.5-Q97.5), Rhats, Bulk ESS and Tail ESS; continuous predictors (age, age^2) were z-transformed; categorical predictor kin relation was dummy coded (reference category unrelated). Credibility intervals not comprising zero are marked in bold.

	Estimate	Est.Error	Q2.5	Q97.5	Rhat	Bulk_ESS	Tail_ESS
Intercept	-0.448	0.308	-1.062	0.167	1	4502.289	6987.839
age_z	0.397	0.381	-0.355	1.136	1	5823.526	7458.165
age_z2	-0.454	0.186	**-0.83**	**-0.104**	1	5687.478	6511.774
target Mother	-2.451	0.525	**-3.54**	**-1.491**	1	10003.627	7587.891
target Kin	-1.077	0.342	**-1.764**	**-0.419**	1	9392.513	8367.582
age_z: target Mother	-5.425	2.13	**-10.108**	**-1.93**	1.001	4259.152	4735.155
age_z: target Kin	-2.188	0.904	**-4.209**	**-0.72**	1.001	6170.922	5766.24
age_z2: target Mother	-4.068	2.577	-9.675	0.192	1.001	4500.148	4879.819
age_z2: target Kin	-0.688	0.974	-2.918	0.81	1.001	6568.416	5898.443

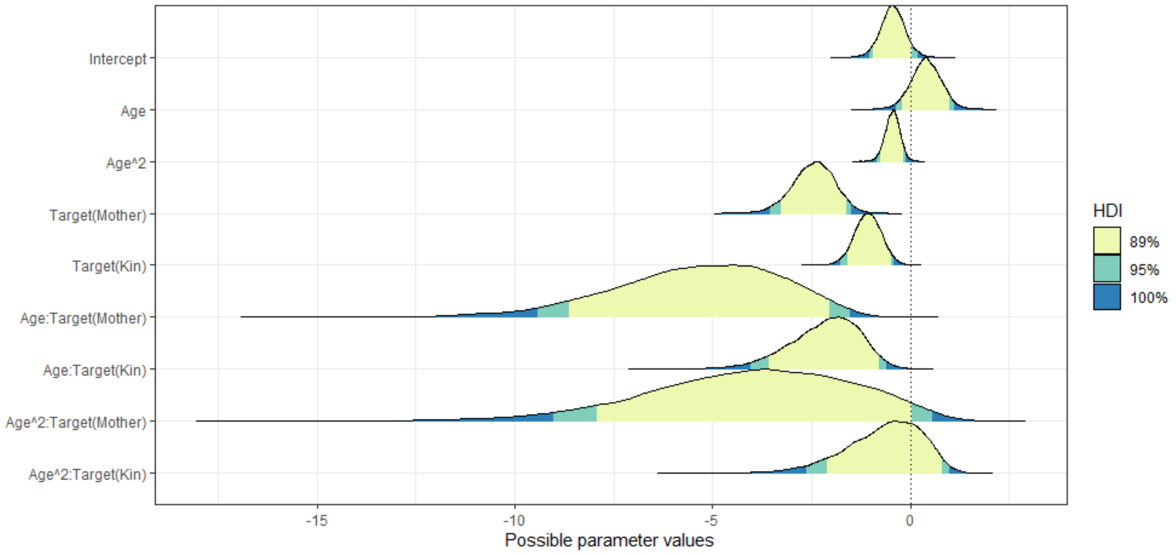

Figure S6: HDI of Model C2, related to Figure 4. HDI are shown for each model estimate with credibility intervals of 89% marked in yellow, of 95% marked in green, and of 100% marked in blue; continuous predictors (age, age^2) were z-transformed; categorical predictor kin relation was dummy coded (reference category unrelated).

Model D: Effect of conspecific dominance rank on peering frequencies

Table S7: Model D output, related to Figure S7. Model estimates and respective standard error, credibility intervals (Q2.5-Q97.5), Rhats, Bulk ESS and Tail ESS; continuous predictors (age, age^2) were z-transformed; categorical predictors rank and sex were dummy coded (reference categories high, female). Credibility intervals not comprising zero are marked in bold.

	Estimate	Est.Error	Q2.5	Q97.5	Rhat	Bulk_ESS	Tail_ESS
Intercept	-1.803	0.37	**-2.541**	**-1.103**	1	9049.655	9557.575
age_z	-2.327	0.508	**-3.396**	**-1.437**	1	8006.156	6642.331
age_z2	-1.786	0.597	**-3.031**	**-0.691**	1.001	7353.677	5850.54
sex male	0.491	0.385	-0.257	1.266	1	9832.883	9079.71
rank low	0.032	0.357	-0.676	0.732	1.001	11381.993	9692.49
rank medium	-0.292	0.298	-0.864	0.303	1	9339.73	9465.277
sex male: rank low	-0.8	0.73	-2.274	0.595	1	11340.272	10143.784
sex male: rank medium	0.671	0.505	-0.315	1.666	1	8732.843	8752.103

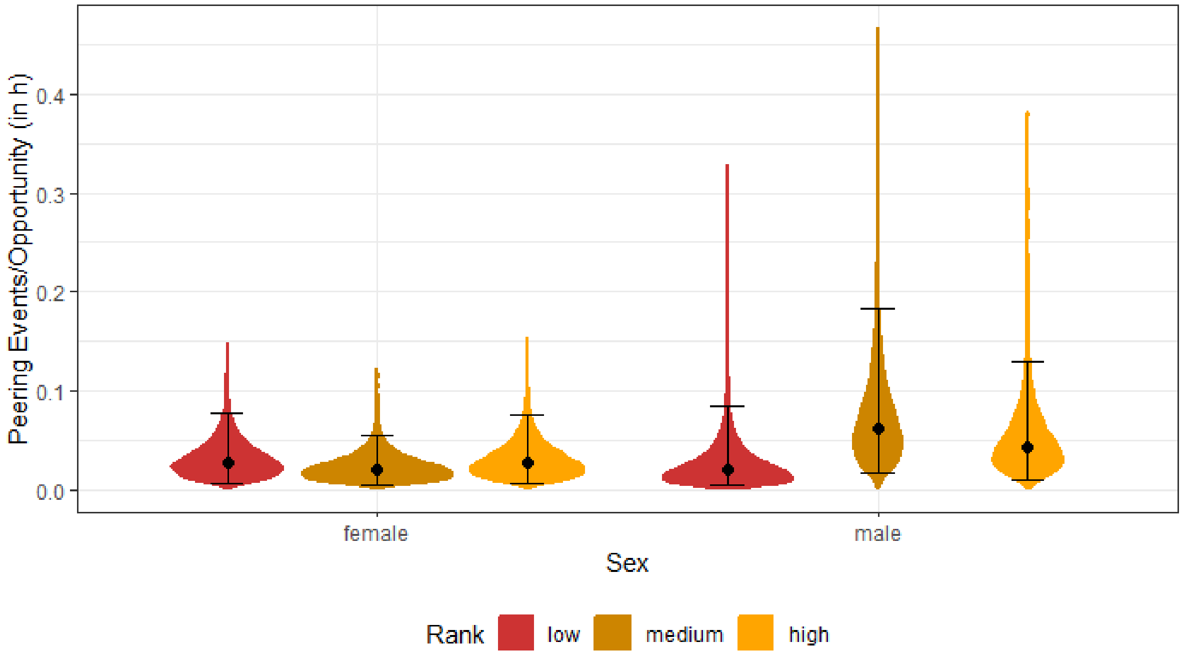

Figure S7: Model Predictions of Model D. Relative peering frequencies directed at low (red), medium (brown), and high (orange) ranking females (left) and males (right).

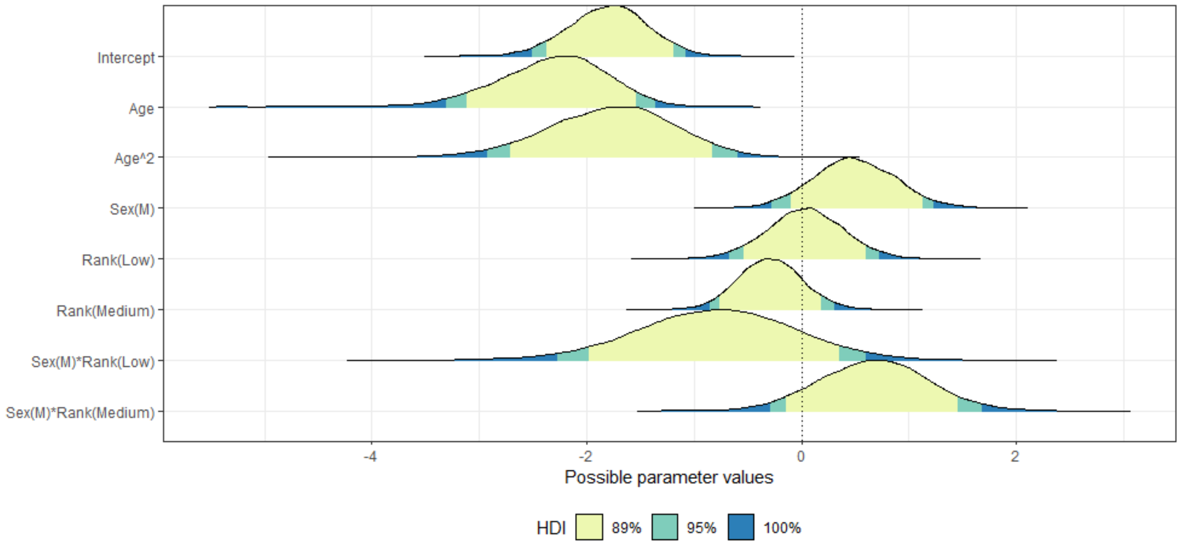

Figure S8: HDI of Model D, related to Figure S7. HDI are shown for each model estimate with credibility intervals of 89% marked in yellow, of 95% marked in green, and of 100% marked in blue; continuous predictors (age, age^2) were z-transformed; categorical predictors rank and sex were dummy coded (reference categories high, female).

Model E: Increased daily grooming rates on peering days

Table S8: Model E output, related to Figure 5. Model estimates and respective standard error, credibility intervals (Q2.5-Q97.5), Rhats, Bulk ESS and Tail ESS; categorical predictor peering was dummy coded (reference category no). Credibility intervals not comprising zero are marked in bold.

	Estimate	Est.Error	Q2.5	Q97.5	Rhat	Bulk_ESS	Tail_ESS
Intercept	-2.167	0.104	**-2.37**	**-1.965**	1.002	1977.584	3640.635
zi_Intercept	-1.427	0.229	**-1.942**	**-1.034**	1	8900.214	10449.209
peerBin2yes	0	0.086	-0.169	0.17	1	14663.57	9898.222
zi_peerBin2yes	-14	10.375	**-41.845**	**-2.933**	1	6132.995	4738.023

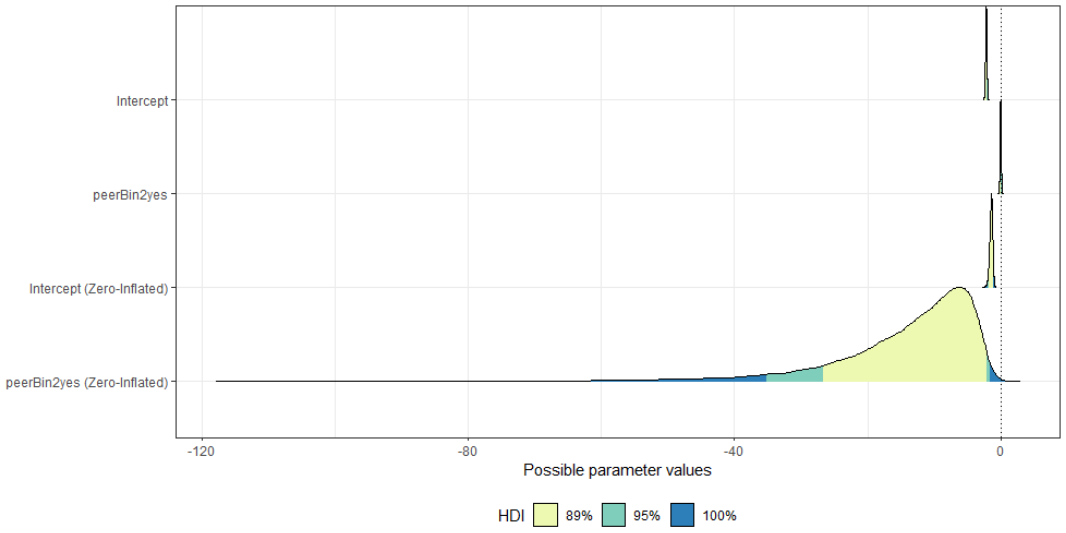

Figure S9: HDI of Model E, related to Figure 5. HDI are shown for each model estimate with credibility intervals of 89% marked in yellow, of 95% marked in green, and of 100% marked in blue; categorical predictor peering was dummy coded (reference category no)

Supplemental references (to Table S9)

1. McGrew, W. C. & Tutin, C. E. Evidence for a social custom in wild chimpanzees? *Man* 13(2), 234-251 (1978).
2. Orangutan Network. Orangutan Data Collection Protocol [Internet]. Available from: https://www.ab.mpg.de/571325/standarddatacollectionrules_suaq_detailed_jan204.pdf
3. Nishida, T. Sexual behavior of adult male chimpanzees of the Mahale Mountains National Park, Tanzania. *Primates* 38, 379-398 (1997).
4. Crockford, C. & Boesch, C. Call combinations in wild chimpanzees. *Behaviour* 142, 397-421 (2005).
5. Zuberbühler, K. Survivor signals: the biology and psychology of animal alarm calling. *Advances in the Study of Behavior* 40, 277-322 (2009).
6. Mori, A. Comparison of the communicative vocalizations and behaviors of group ranging in eastern gorillas, chimpanzees and pygmy chimpanzees. *Primates* 24, 486-500 (1983).
7. Shumaker, R. W., Walkup, K. R. & Beck, B. B. *Animal tool behavior: the use and manufacture of tools by animals*. JHU Press (2024).
8. Whiten, A. *et al*. Charting cultural variation in chimpanzees. *Behaviour* 138, 1481-1516 (2001).
9. Goodall, J. Tool-using and aimed throwing in a community of free-living chimpanzees. *Nature* 201(4926), 1264-1266 (1964).
